# Role of 25 MHz Ultrasound Biomicroscopy in the Detection of Subluxated Lenses

**DOI:** 10.1155/2018/3760280

**Published:** 2018-10-17

**Authors:** Mingyu Shi, Liwei Ma, Jinsong Zhang, Qichang Yan

**Affiliations:** Department of Ophthalmology, The Fourth Affiliated Hospital of China Medical University, Eye Hospital of China Medical University, The Key Laboratory of Lens in Liaoning Province, Shenyang, 110005 Liaoning, China

## Abstract

**Background:**

The purpose of this observational case series study was to investigate the role of 25 MHz ultrasound biomicroscopy (UBM) in detecting subluxated lenses and compare it with 50 MHz UBM.

**Methods:**

45 patients (49 eyes) with suspected subluxation of the lens and 20 normal volunteers (40 eyes) were included. Different cross-sectional images of the lens position were captured in axial and longitudinal scanning modes using 25 and 50 MHz UBM. The main outcome measurements included the linear distance between the lens equator and ciliary process, the difference value (D-value) between the same cross section of the above bilateral linear distance in the normal and the subluxated subjects, the diagnostic accuracy, and the testing times obtained with 25 and 50 MHz UBM.

**Results:**

The position of the lens on axial sections could be clearly shown by using 25 MHz UBM. The D-value of the subluxated eyes was 1-2 mm longer than that of the normal ones. There was a statistically significant difference between 25 and 50 MHz UBM in showing subluxation of the lens, the testing time was significantly faster (2.0 min versus 7.5 min), and the diagnostic accuracy was much higher (98.0% versus 71.4%) with 25 versus 50 MHz UBM. Fifteen eyes with slightly subluxated lens were detected by 25 MHz UBM, and only one eye with slight lens subluxation was detected by 50 MHz UBM.

**Conclusions:**

The results indicated that 25 MHz UBM has a greater diagnostic value than 50 MHz UBM in verifying the status of the lens subluxation and can provide reliable and quantitative imaging evidence for clinical use. This trial is registered with ChiCTR–DOD –15007603.

## 1. Introduction

Subluxation of the lens is a common lens disease, and most cases require surgical treatment. Accurate preoperative localization of the lens is necessary for surgical planning. Complete luxation of the lens can be easily detected by low-frequency (5–14 MHz) B-scan ultrasound [1, 2], a slit lamp, or an ophthalmoscope. However, subluxation of the lens, especially slight subluxation of the lens, cannot be determined directly by the above means. Most subluxated cases as well as other lens-related diseases, such as cataracts, glaucoma, and ocular trauma, require surgical treatment. Accurate localization of the lens is important to determine a reasonable operation plan to avoid intraoperative and postoperative complications. Therefore, it is essential to accurately diagnose subluxation of the lens preoperatively.

Ultrasound biomicroscopy (UBM), a high-frequency (50–100 MHz), high-resolution imaging technique, offers cross-sectional images of the anterior segment to a depth of 5 mm [3–5] and improves the detection rate of subluxated lenses. Compared to 50 MHz UBM, 25 MHz UBM has a relatively low frequency, which is focused on the lens to a scanning depth of 9 mm. It is worth discussing whether any advantage exists in examining subluxated lenses using 25 MHz UBM. However, to the best of our knowledge, information regarding these two different UBM frequencies is scarce. This study discusses these two different imaging modalities and their role in the diagnosis of subluxated lenses.

## 2. Participants and Methods

### 2.1. Participants

A total of 20 normal volunteers (40 eyes) and 45 suspected cases (49 eyes) of lens subluxation were selected, including 26 females (40.0%) and 39 males (60.0%), from February 2017 to October 2017. Participants' ages ranged from 21 to 85 years (mean ± SD: 51.4 ± 14.4 years). The normal eye criteria included the logarithmic minimal angle resolution (logMAR), uncorrected visual acuity (UCVA) was 0.8–1.0, the slit lamp examination was normal, and without ocular history. Among the suspected subluxated ones, 19 eyes (38.8%) exhibited age-related cataracts, 17 eyes (34.7%) exhibited traumatic cataracts, 8 eyes (16.3%) exhibited cataracts after glaucoma surgery, 4 eyes (8.2%) exhibited congenital subluxation of the lens, and 1 eye (2%) exhibited a ciliary body tumor.

### 2.2. Methods of Examination

All subjects underwent 50 MHz panoramic UBM (MD-300L, MEDA Co., Ltd., Tianjin, China) examinations firstly, then underwent 25 MHz panoramic UBM (MD-320, MEDA Co., Ltd., Tianjin, China) examinations after ten minutes of rest with the same gain (75 dB), indoor light, and watching distance (33 cm) to capture axial and longitudinal section images of the lens at the Eye Department of the Fourth Affiliated Hospital of China Medical University (Shenyang, Liaoning, China) by an experienced examiner *(S.M.Y)*. These two UBM scanners allowed 4-5 mm and 9-10 mm tissue penetration and axial resolution of approximately 50 *μ*m, 9-10 mm tissue penetration was used in the axial scan, and 4-5 mm tissue penetration was used in the longitudinal scan. In both 25 and 50 MHz UBM, the axial and lateral resolutions were no greater than 0.05 mm and 0.1 mm, and the axial and lateral geometric position accuracies were no greater than 10% and 15%. An immersion B-scan technique was employed, and the patients were placed in a supine position after superficial anesthesia. A sterile scleral cup was placed into the conjunctival sac and filled with distilled water as a coupling agent.

In the normal subjects, one vertical axial section (12:00–6:00) and eight longitudinal sections (12:00, 1:30, 3:00, 4:30, 6:00, 7:30, 9:00, and10:30) images were captured by 50 MHz UBM, while four axial sections (12:00–6:00, 3:00–9:00, 4:30–10:30, and 7:30–1:30) images were captured by 25 MHz UBM. In the subluxated subjects, the longitudinal scans were mainly used in 50 MHz UBM to determine the range and the most significant direction of lens subluxation carefully, and the axial scans were mainly used in 25 MHz UBM to determine the subluxated lens. Three clear images in the most significant subluxated direction of these two UBMs were captured to measure the parameters of the lens position by the same operator *(S.M.Y)*.

### 2.3. Parameter Measurements

The average linear distance between the lens equator and ciliary process, the difference value (D-value) between the same cross section of the above bilateral linear distance in the normal subjects and the lens subluxation in the most significant direction, the diagnostic accuracy, and the testing times obtained with 25 and 50 MHz UBM were compared.The distance values were the average of three measurements according to the images obtained with 25 and 50 MHz UBM. All subjects were fully informed of the details and possible risks of the examination, and written informed consent was obtained from all patients before examination following the tenets of the Declaration of Helsinki. The study was approved by the Ethics Committee of the Fourth Affiliated Hospital of China Medical University and was registered at http://www.chictr.org.cn (study registration no: ChiCTR–DOD–15007603).

### 2.4. Data and Statistical Analysis

The statistical analysis was performed using SPSS 19.0 for Windows (SPSS, Inc., IBM, Armonk, New York, USA), and the results are presented as the means (±SD) along with 95% confidence intervals (CIs). We used independent samples *t*-tests and chi-square tests to analyze the differences. A value of *P* < 0.05 was considered significant.

## 3. Results

### 3.1. Image Features of These Two UBMs

**Figure 1:**
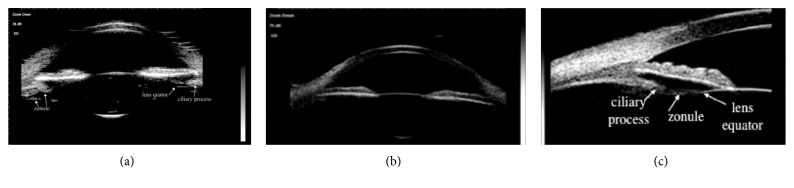
The lens position images of normal subjects obtained in the same patient with 25 and 50 MHz UBM. (a) The normal subject image of 25 MHz UBM in the axial section, from left to right, the ciliary process, lens equator, and zonule were marked by arrows; (b) the normal subject image of 50 MHz UBM in the axial section; (c) the normal subject image of 50 MHz UBM in the longitudinal section, the ciliary process, zonule, and lens equator were marked by the arrows from left to right.

The lens position images in normal subjects obtained with 25 MHz and 50 MHz UBM were shown in Figure 1. The axial section image of 25 MHz UBM revealed that the smooth arc lines in strong echo of anterior and posterior capsule of the lens were located in the center of the pupil area, the zonule was visible as a thin and weak stripe-like echo attached to the ciliary process, and the linear distances between lens equator and ciliary process in all directions were equal (Figure 1). On the 50 MHz UBM axial section image, the echoes of the lens were weaker than those of 25 MHz UBM, and the zonule was barely visible (Figure 1). In the longitudinal section of 50 MHz UBM, the relationship between lens equator and its surrounding anterior segment tissue was shown clearly (Figure 1).

**Figure 2:**
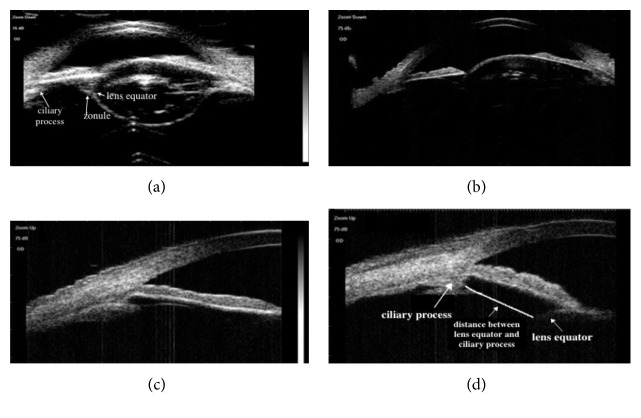
Subluxated lenses with age-related cataract images obtained in the same patient with 25 and 50 MHz UBM. (a) A 25 MHz UBM axial section image shows that the anterior chamber geometry is distorted, the distance between the ciliary process and lens equator is increased on the subluxated side, and the side of the lens equator with increased distance is more curved than the other side; the opacity of the lens, ciliary process, lens zonule, and lens equator are shown clearly (arrows); (b) a 50 MHz UBM axial section image shows only that the anterior chamber geometry is distorted; the distance of the subluxated side and other lesion features of the lens are not shown clearly; (c, d) the 50 MHz UBM longitudinal section images show the difference in the distance between the ciliary process and lens equator. The distance between the ciliary process and lens equator in (d) is longer than that in (c).

**Figure 3:**
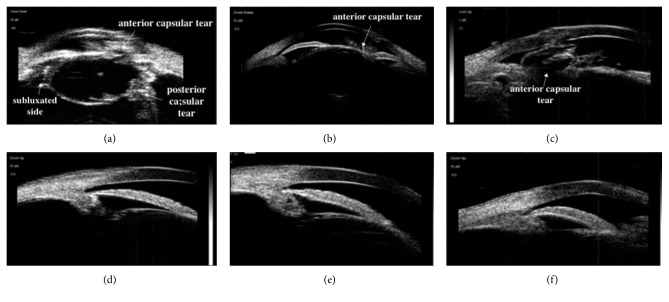
Slightly subluxated lens with traumatic cataract images obtained in the same patient with 25 and 50 MHz UBM. (a) A 25 MHz UBM image clearly shows a slightly subluxated lens, the anterior and posterior capsular tear of the lens (arrows), and the opacity of whole lens; (b) an axial image of 50 MHz UBM shows only the tear and opacity of the anterior lens capsule (arrow); other lens lesions were not shown clearly, especially in the posterior capsule of the lens; and the tear location could not be seen; (c–f) the longitudinal section images of 50 MHz UBM show only the tear and opacity of the anterior lens capsule (arrow), and the differences in the distance between the lens equator and the ciliary process in these figures were not significantly different.

The diagnosis of a subluxated lens using UBM was made based on the difference in the linear distance between the lens equator and ciliary process in every direction; the side of the lens equator with a greater distance was more curved than the other side. These image features could be directly obtained with 25 MHz UBM in axial sections (Figures 2 and 3). Particularly in cloudy lenses, 25 MHz UBM not only displayed the subluxated lens image features intuitively but also displayed the lens opacity morphology (Figure 2), and the integrity of the lens posterior capsule could be shown clearly (Figure 3). These lesions could not be clearly viewed in axial sections of 50 MHz UBM (Figures 2 and 3). Determination of the difference in the distance between the lens equator and ciliary process was obtained only by comparison among various longitudinal sections of 50 MHz UBM (Figures 2–2 and 3–3). The whole lens opacity morphology and the integrity of the posterior capsule of the lens were not obtained in either axial or longitudinal sections in 50 MHz UBM.

### 3.2. Comparison of the Normal and the Subluxated Parameters between These Two UBMs

The measurement results of the normal and the subluxated subjects obtained with 25 and 50 MHz UBM were shown in Table 1. By comparison, in the normal subjects, the distance between lens equator and the ciliary process in all cross sections has no difference, so the average distance and the D-value of the normal subjects were used to make the comparison. Except the D-value of the normal subjects which had no significant difference between 25 and 50 MHz UBM, the statistically significant differences were observed between the normal subjects and the subluxated lens, between 25 and 50 MHz UBM for the subluxated lens parameters (Table 1).

Forty-eight eyes were diagnosed as subluxated lens by 25 MHz UBM, and the diagnostic accordance rate of lens subluxation with intraoperative findings was 98.0%. Thirty-five eyes were diagnosed by 50 MHz UBM, and the diagnostic accordance rate was only 71.4%. A significant difference was observed in the diagnostic accordance rate between 25 and 50 MHz UBM. The diagnostic accuracy of 25 MHz UBM was significantly higher than that of 50 MHz UBM (Table 2).

### 3.3. Slight Subluxation of Lens

The results showed that 25 MHz UBM detected 15 eyes with slight subluxation of the lens, which were not clinically diagnosed as subluxation of the lens, while only 1 eye among these 15 eyes was diagnosed with subluxation of the lens by 50 MHz UBM. The significant difference was observed among normal lens position and slight and other subluxations of the lens detected by 25 MHz UBM except the minimum distance between lens equator and ciliary process of the subluxated lens (Table 3).

## 4. Discussion

Under normal physiological conditions, the anatomical location of the lens is behind the iris and fixed in the ciliary process by the zonule [6], so the radians of the lens equator are consistent and there is almost no difference in distance between lens equator and ciliary process in the same cross section. Our study has also confirmed this fact by using 25 and 50 MHz UBM to observe the normal subjects. Ocular trauma, congenital causes, cataracts, or other factors may cause rupture of the zonule, which leads to subluxation of the lens [7]. When the lens is subluxated, the lens equator of the subluxated side appears more curved than that of the side with an intact zonule. This is due to the elastic properties of the lens [8]; without the traction of the zonule, the lens could be retracted and relies on its own flexibility. At the same time, the subluxated lens sinks due to gravity; therefore, the linear distance between the lens equator and ciliary process is increased on the subluxated side. 25 MHz UBM showed these morphological features of subluxated lenses intuitively in axial sections in our study. Because 25 MHz UBM has a relatively low frequency and high penetration and the ultrasound focus is on the lens, the lens and its surrounding tissues can be displayed comprehensively in axial sections. Therefore, during examination, the patients need only to look straight ahead without moving their eyes, which can relieve the suffering of patients and shorten the testing time. Our study showed that the testing time of 25 MHz UBM was only 1/3 of that of 50 MHz UBM. Compared to 25 MHz UBM in axial sections, 50 MHz UBM can hardly show the zonule and equators of lens [9]; therefore, the subluxation of the lens is not diagnosed accurately, except for obvious dislocation with a distortion of anterior chamber geometry. To obtain a distinct image of the lens equator and its surroundings, patients must move their eyes substantially in longitudinal sections. Meanwhile, the eye cup embedded in the conjunctival sac limits the eye rotation to some extent, and some patients are unable to cooperate, especially in the superior and inferior directions; as a result, the eyes cannot reach the ideal position. All of these factors may cause the testing time to be extended and reduce the diagnostic rate.

In this study, although the lens images of these two types of UBM are at different magnifications, the measurement software for these two devices can adjust the scale automatically according to different image magnifications, which ensures that the measurement results are consistent with different image magnifications in the same anterior segment of the same patient. Therefore, the differences in the measurements resulting from the different magnifications of these two UBM images are negligible. Moreover, in order to reduce the measurement errors that were led by morphological changes and uncomfortable feeling because of long-time constriction in the eyes with an eyecup, every subject had a ten minutes rest between the two UBMs examination. And all subjects were in the same indoor environment and watching distance during examination, as well as one skill operator performed all examinations and measurement, which ensure the consistency of all inspection conditions. The reason for different measurement results of the normal subjects between the two UBMs may have the relationship with the eye position. The longitudinal scan mode of 50 MHz UBM requires the patient to move the eyes, which may cause the tense state of the eyes and the change of the positional relationship between the lens equator and the ciliary process. Compared with the longitudinal section, the eyes are relatively relaxed and static at the axial scanning mode. Another reason may be that the poor rotation of the eyes and patient tolerance reduce the definition of image in 50 MHz UBM and cause the measurement error. 25 MHz UBM can show the lens and its surrounding anterior segment tissue clearly and intuitively in the axial section, so we think that the measurements obtained with 25 MHz UBM may be more reliable compared with 50 MHz UBM.

The different measurement results for the subluxated lens indicated that 25 MHz UBM was more sensitive than 50 MHz UBM in evaluating the position of the lens. In the present study, the diagnosis rate of 25 MHz UBM was 98.0%; only 1 eye was not diagnosed by 25 MHz UBM; and because the patient had a serious ocular blunt trauma ten days earlier, he could not cooperate with the examination. Compared to 25 MHz UBM, the diagnostic rate of 50 MHz UBM was only 71.4%. More importantly, 25 MHz UBM has a more significant advantage for displaying slightly subluxated lenses. According to the present results, slightly subluxated lenses should be carefully considered in patients, as this type of case is easily misdiagnosed in clinical practice due to a lack of obvious iris tremor [10]. However, these cases can be diagnosed by modern imaging techniques or intraoperatively because the distance between the lens equator and ciliary process on the subluxated side is less than 1.5 mm. The 25 MHz UBM images showed that 15 eyes had slight lens subluxation, and all were confirmed intraoperative. It can be seen that the 25 MHz UBM has high sensitivity in judging the lens position, and even the slight difference in the range of 0.5 to 1.0 mm can be shown. In contrast, 50 MHz UBM images revealed only 1 eye with slight lens subluxation of these 15 eyes. During the examinations, we also found that the subluxated lens positions and ranges of the same patients diagnosed by these two UBM frequencies were the same. Previous researchers compared direct and indirect methods to diagnose slight lens dislocation using 50 MHz UBM [11]; the indirect method measured the vertical distance between the edge of the pupil and the surface of the lens in axial sections. These studies noted that axial scanning was a time-saving, simple, and effective method, but they also emphasized that this method was greatly affected by pupil size. Our study could accurately determine the location of the lens in axial sections without the influence of pupil size using 25 MHz UBM. Therefore, we propose that 25 MHz UBM examinations should be included in routine preoperative examinations of all ophthalmic diseases requiring lens location determination.

Additionally, 25 MHz UBM cannot only show the accurate location of the lens but can also display the integrity of the posterior lens capsule, which is particularly important for traumatic cataracts. Preoperative determination of the presence of an intact posterior lens capsule through echographic confirmation assists the physician in surgical planning [12]. In our study, a tear of the posterior lens capsule was found in 6 of the 17 eyes with traumatic cataracts using 25 MHz UBM. In comparison, 50 MHz UBM allows, at best, visualization of the anterior lens capsule only due to its high resolution but lack of penetration [3]; 2 of these 6 eyes had a tear of the anterior capsule, and rupture of the posterior capsule was not shown by 50 MHz UBM. Previous studies have used 20 MHz B scans to observe the posterior capsule of the lens [13, 14]. A.Tabatabaei et al. [14] found 93% sensitivity and 86% specificity for the ability of a 20 MHz probe to detect a posterior capsule tear of any size. M. Kucukevcilioglu et al. [15] reported one case with a 1.0 mm posterior capsule irregularity using 35 MHz UBM and emphasized that 35 MHz UBM was equipped with 70 *μ*m of axial-lateral resolution and penetration with a 7.0 to 8.0 mm probe and could provide more detailed evaluation of the zonule and ciliary body because of its deeper penetration than 50 MHz UBM as well as higher resolution than 20 MHz B scans. However, 25 MHz UBM with a penetration of 9.0 mm and 50 *μ*m of axial-lateral resolution is more suitable for displaying the posterior capsule integrity and other lens lesions.

At present, some optical devices, such as Scheimpflug imaging and anterior segment optical coherence tomography (AS-OCT), have been used to study the morphology of the anterior segment [16, 17]. Although these two devices can provide accurate and objective measurements of anterior segment parameters, they are still affected by the size of the pupil and the clarity of the refractive media; it is difficult to visualize the posterior cortex and posterior capsule, even if the pupil is fully dilated [18–20]. Considering the limitations in observing the positional relationship between the lens and its surrounding tissues using optical devices, this study did not compare them with ultrasonic equipment. Panoramic 25 MHz UBM can show equators of the lens and the ciliary process synchronously without mydriasis, which can accurately and intuitively determine whether lens subluxation exists; therefore, 25 MHz UBM is more suitable for the observation of subluxated lenses than optical devices.

## 5. Conclusion

25 MHz UBM has great diagnostic value in verifying the status of the lens location, and it provides accurate and reliable information about the status of lens subluxation beyond 50 MHz UBM. Additionally, 25 MHz UBM is a useful and novel approach for diagnosing lens subluxation, and the combination of image and measuring data will maximize the detection rate of lens dislocation. We believe that, with the development of ultrasound technology, an increasing number of different frequencies of ultrasonic equipment will be applied in ophthalmology.

## Figures and Tables

**Table 1:** The measurements of lens position in normal and subluxated subjects obtained with 25 MHz and 50 MHz UBM.

Measurements	25 MHz UBM	50 MHz UBM	*P*
*The normal eyes*			
Distance (mm)	1.01 ± 0.20	0.83 ± 0.20	0.001
D-value (mm)	0.01 ± 0.01	0.01 ± 0.01	1.678
*The subluxated eyes*			
Maximum distance (mm)	2.00 ± 0.84	1.64 ± 0.91	0.001
Minimum distance (mm)	0.42 ± 0.41	0.59 ± 0.52	0.029
D-value (mm)	1.54 ± 0.78	1.02 ± 0.74	0.000
Testing time (min)	2.05 ± 0.34	7.48 ± 2.53	0.000
*Normal* versus *subluxated eyes*	0.000	0.000	

All measuring values are presented as mean ± SD along with 95% confidence intervals (CIs). Distance = the linear distance between the lens equator and the ciliary process; D-value = the difference of the bilateral distance between lens equator and the ciliary process in the same cross section.

**Table 2:** Diagnostic accuracy comparison between 25 MHz and 50 MHz UBM.

	25 MHz UBM	50 MHz UBM	*X* ^2^	*P* ^*∗*^
Confirmed eyes (*n*)	48	35	8.611	0.003
Undiagnosed eyes (*n*)	1	14		

*P* < 0.05.

**Table 3:** Comparison among normal subjects and slight and other subluxations of 25 MHz UBM.

Distance (mm)	Normal subjects (*n*=40)	Slight subluxation (*n*=15)	Other subluxations (*n*=15)	*P*
Maximum	1.01 ± 0.20	1.12 ± 0.29	2.06 ± 0.81	0.000^*∗*^
Minimum		0.38 ± 0.29	0.41 ± 0.49	0.800
D-value	0.01 ± 0.01	0.76 ± 0.27	1.58 ± 0.76	0.000^*∗*^

All measuring values are presented as means ± SD along with 95% confidence intervals (CIs). Maximum = the maximum distance between lens equator and ciliary process of the subluxated lens; minimum = the minimum distance between lens equator and ciliary process of the subluxated lens; D-value = the difference between maximum and minimum distance of the subluxated lens. ^*∗*^*P* < 0.05.

## Data Availability

All data of this article can be obtained from the corresponding author and the first author.
